# Supplementary material for: AMPKα1 Regulates Lung and Breast Cancer Progression by Regulating TLR4-Mediated TRAF6-BECN1 Signaling Axis
Source: Cancers (Basel). 2020 Nov 6;12(11):3289. doi: 10.3390/cancers12113289 (PMC7694660; doi:10.3390/cancers12113289)
Supplement: Supplementary file 1 [file cancers-12-03289-s001.pdf]

Supplementary Materials: AMPKα1 regulates lung and breast cancer progression by regulating TLR4-mediated TRAF6-BECN1 signaling axis

Mi-Jeong Kim, Yoon Min, Juhee Son, Ji Young Kim, Ji Su Lee, Duk-Hwan Kim and Ki-Young Lee

1. Supplementary materials and methods

1.1. Construction of truncated mutants

Truncated mutants of FLAG-TRAF6, FLAG-AMPKαl, and MYC-BECN1 were generated, as previously described [1–6].

1.2. AMPKα1-knockout cells

Guide RNA sequences for CRISPR/Cas9 were designed and AMPKα1KO HEK293T, *AMPKαl*KO MDA-MB-231, and *AMPKαl*KO MCF-7 cells were generated, as previously described [2,4,7]. Insert oligonucleotides for human *AMPKαl* gRNA were 5′- CACCGGAAGATTCGGAGCCTTGATG -3′/3′-CCTTCTAAGCCTCGGAACTACCAAA -5′. The complementary oligonucleotides for guide RNAs (gRNAs) were annealed, and cloned into lenti CRISPR v2 vector (Addgene plasmid, Ca#52961). Lenti CRISPR v2/gRNA was transfected into HEK293T cells by using Lipofectamine 2000, according to the manufacturer’s instructions. *AMPKαl*KO colonies were isolated, as previously described [2,4,7], and confirmed by using western blot.

1.3. Western blotting analysis and endogenous immunoprecipitation assay

Cell lysates from control (Ctrl) HEK293T and *AMPKαl*KO HEK293T cells, Ctrl MDA-MB-231 and *AMPKαl*KO MDA-MB-231, or Ctrl MCF-7 and *AMPKαl*KO MCF-7 cells were separated by 6–10% SDS-PAGE and probed with anti-AMPKαl or anti-GAPDH antibody. Ctrl MDA-MB-231 and *AMPKαl*KO MDA-MB-231, or Ctrl MCF-7 and *AMPKαl*KO MCF-7 cells were treated with or without 3-MA (5mM), or CQ (10 μM), in the presence or absence of LPS (10 μg/ml), for 6 h. Whole cell lysates were immunoblotted with anti-LC3 antibody and anti-GAPDH as a loading control. For endogenous immunoprecipitation assay, A549 cells (5×10^7 cells per sample) were treated with or without LPS (10 μg/ml) for 1 h. Cells were harvested and lysed on ice for 1 h with addition of 1.0 mL ice-cold RIPA buffer. The cellular debris were pelleted by centrifugation at 10,000 rpm for 10 min at 4 °C. The supernatant was then transferred to a fresh conical centrifuge tube on ice. Primary antibody (anti-TRAF6,1:200) and IgG antibody as a control were added and incubated for 4 h at 4 °C. Then, protein A/G PLUS-Agarose was added and incubated at 4 °C on a rocker platform for 4 h. The immuneprecipitate was collected by centrifugation at 2500 rpm for 5 min at 4 °C. The pellet was then washed four times with 1.0 mL RIPA buffer. After the final wash, the pellet was resuspended in 100 μL of 3% SDS. The samples were boiled for 5 min, and 20 μL aliquots were separated by SDS-PAGE and probed with anti-TRAF6, anti-BECN1, or anti-AMPKαl antibody.

1.4. Reverse Transcription-Quantitative Polymerase Chain Reaction (RT-qPCR)

RT-qPCR analysis was performed, as previously described [4]. Briefly, Control (Ctrl) and *AMPKαl*KO A549 cells were treated without, or with 10 μg/mL LPS or LPS plus 3-MA (5 mM) for 6 h. Total RNA was extracted from cells using an RNA isolation kit (A&A Biotechnology, Gdynia, Poland) according to the manufacturer’s protocol. cDNA was obtained by RT using a amfiRivert II cDNA Synthesis Master Mix (genDEPOT, R550), according to the manufacturer’s protocol. Primers for hIL-6 (PPH 00560C), hMMP2 (PPH 00151B), and hCCL2 (PPH 00192F) were purchased from Qiagen, Inc. (Chatsworth, CA, USA). Fluorescence detection was performed using the ABI PRISM

7700 Sequence Detector (PerkinElmer; Applied Biosystems; Thermo Fisher Scientific, Inc.). The mRNA expressions were calculated and normalized to the level of GAPDH.

1.5. Correlation analysis of AMPKα1 (PRKAA1) in lung cancers

To analysis the correlation between AMPKαl (*PRKAA1*) and TRAF6, TAK1 (*MAP3K7*), or TLR4 in lung cancers, we used GEPIA (Gene expression profiling interactive analysis, http://gepia.cancer-pku.cn/).

2. Supplementary Tables

Table 1. Characteristics of the patients.

Patient ID	Age	Sex	Histology	Stage[a]
LT01	76	Man	Squamous cell carcinoma	IIB
LT02	77	Man	Squamous cell carcinoma	IIB
LT03	75	Man	Large cell carcinoma	IIA
LT04	65	Man	Large cell carcinoma	IIB
LT05	67	Man	Squamous cell carcinoma	IIIA
LT06	67	Man	Squamous cell carcinoma	IB
LT07	64	Man	Large cell carcinoma	IIA
LT08	59	Man	Squamous cell carcinoma	IIB
LT09	65	Woman	Adenocarcinoma	IB
LT10	53	Woman	Adenocarcinoma	IIA
LT11	54	Woman	Adenocarcinoma	IB
LT12	56	Woman	Adenocarcinoma	IB
LT13	70	Man	Adenocarcinoma	IA
LT14	68	Woman	Adenocarcinoma	IB
LT15	72	Woman	Large cell carcinoma	IB
LT16	69	Man	Pleomorphic carcinoma	IA
LT17	69	Man	Adenocarcinoma	IA
LT18	60	Woman	Adenocarcinoma	IB
LT19	84	Man	Adenocarcinoma	IB
LT20	70	Man	Adenocarcinoma	IIB
LT21	59	Man	Adenocarcinoma	IB
LT22	38	Woman	Adenocarcinoma	IIA
LT23	77	Man	Adenocarcinoma	IA
LT24	79	Woman	Adenocarcinoma	IB
LT25	47	Woman	Adenocarcinoma	IB
LT26	81	Man	Adenocarcinoma	IB
LT27	66	Woman	Adenocarcinoma	IIA
LT28	75	Man	Adenocarcinoma	IA
LT29	65	Man	Adenocarcinoma	IA
LT30	50	Woman	Adenocarcinoma	IIA
LT31	70	Man	Adenocarcinoma	IB
LT32	72	Man	Large cell carcinoma	IIA
LT33	68	Man	Adenocarcinoma	IB
LT34	59	Woman	Adenocarcinoma	IIA
LT35	70	Man	Adenocarcinoma	IB
LT36	63	Man	Squamous cell carcinoma	IB
LT37	68	Man	Adenocarcinoma	IIB
LT38	67	Man	Squamous cell carcinoma	IIB
LT39	72	Man	Squamous cell carcinoma	IB
LT40	73	Woman	Adenocarcinoma	IB
LT41	43	Woman	Adenocarcinoma	IB
LT42	64	Man	Large cell carcinoma	IB

[a]The 7th edition of the TNM classification for lung cancer

3. Supplementary Figures

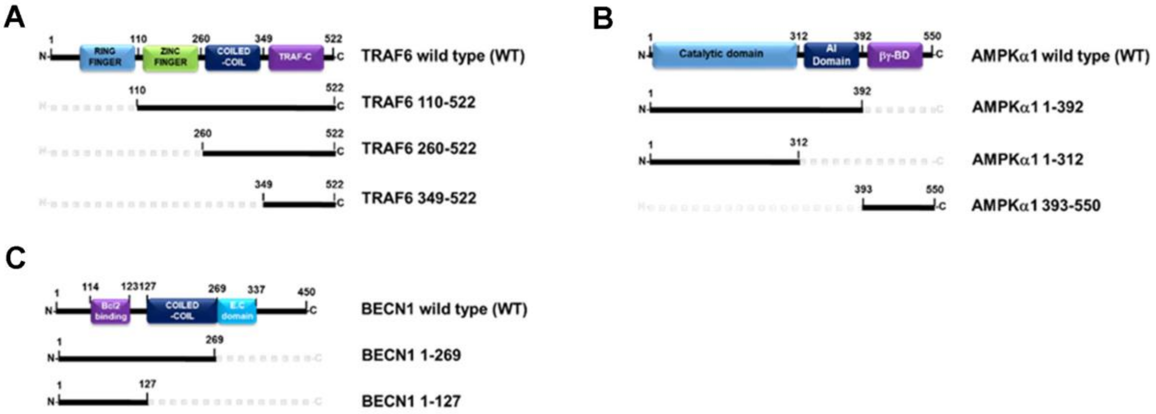

Figure S1. Truncated mutants of TRAF6, AMPKα1, and BECN1. (**A**) Truncated mutants of TRAF6, TRAF6 110-522, TRAF6 260-522, and TRAF6 349-522 were generated, as described in supplementary Materials and Methods. (**B**) Truncated mutants of AMPKα1, AMPKα1 1-392, AMPKα1 1-312, and AMPKα1 393-550 were generated, as described in supplementary Materials and Methods. (**C**) Truncated mutants of BECN1, BECN1 1-269 and BECN1 1-127 were generated, as described in supplementary Materials and Methods.

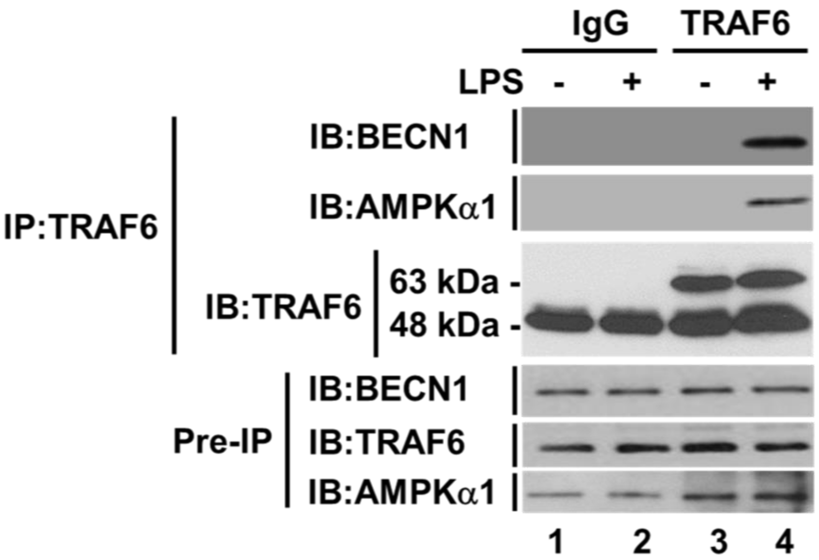

Figure S2. Endogenous immunoprecipitation assay with anti-TRAF6 antibody in A549 lung cancer cells. A549 cells (5×10^7 cells per sample) were treated with or without LPS (10 mg/ml) for 1 h. Cells were harvested and lysed on ice for 1 h with addition of 1.0 mL ice-cold RIPA buffer. The cellular debris were pelleted by centrifugation at 10,000 rpm for 10 min at 4 °C. The supernatant was then transferred to a fresh conical centrifuge tube on ice. Primary antibody (anti-TRAF6,1:200) and IgG antibody as a control were added and incubated for 4 h at 4 °C. Then, protein A/G PLUS-Agarose was added and incubated at 4 °C on a rocker platform for 4 h. The immunoprecipitate was collected by centrifugation at 2500 rpm for 5 min at 4 °C. The pellet was then washed four times with 1.0 mL RIPA buffer. After the final wash, the pellet was resuspended in 100 µL of 3% SDS. The samples were boiled for 5 min, and 20 µL aliquots were separated by SDS-PAGE and probed with anti-TRAF6, anti-BECN1, or anti-AMPKal antibody. Uncropped western blot images available in Supplementary Figure S12.

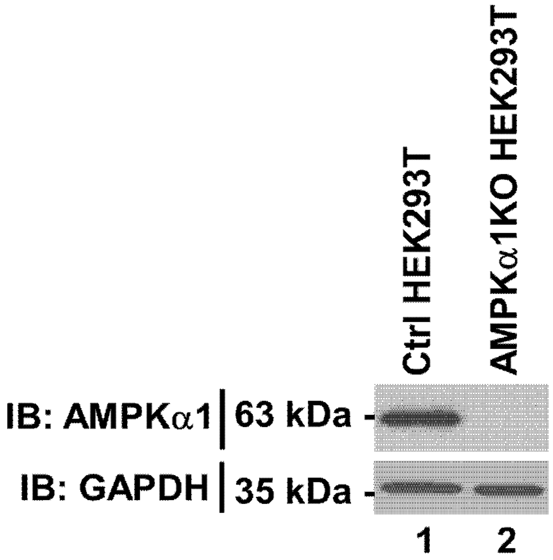

Figure S3. Generation of *AMPKα1*-knockout HEK293T cells. *AMPKα1*-knockout HEK293T cells were generated, as described in supplementary Materials and Methods. The efficacy of AMPKα1-knockout was examined by using western blotting with antibodies to AMPKα1 or GAPDH as loading control. Uncropped western blot images available in Supplementary Figure S13.

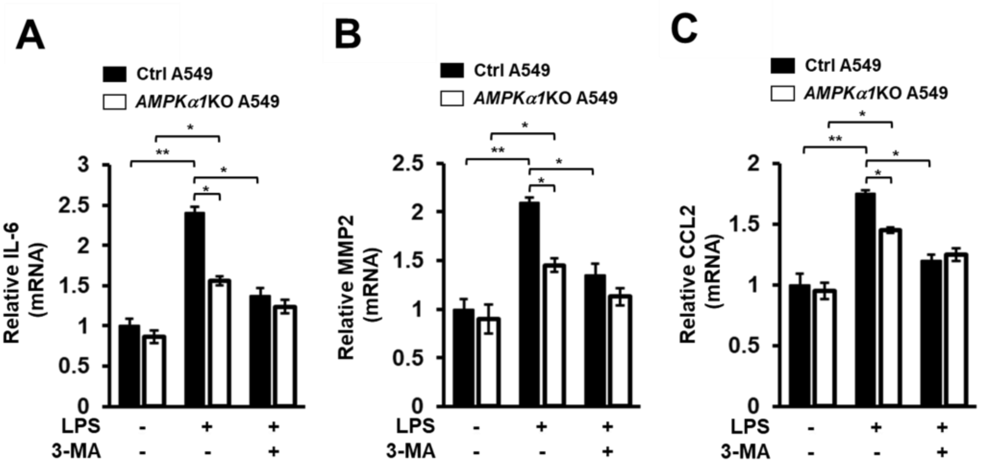

Figure S4. Measurement of IL-6 mRNA, MMP2 mRNA, and CCL2 mRNA in Ctrl A549 and AMPKα1KO A549 cells in response to LPS stimulation. Control (Ctrl) and *AMPKαl*KO A549 cells were treated without, or with 10 μg/mL LPS or LPS plus 3-MA (5 mM), as indicated. Total RNA was extracted, cDNA was obtained, as described in supplementary Materials and Methods, and RT-qPCR analysis performed with specific primers, such as hIL-6 (**A**), hMMP2 (**B**), and hCCL2 (**C**). *$p < 0.05$, **, $p < 0.01$.

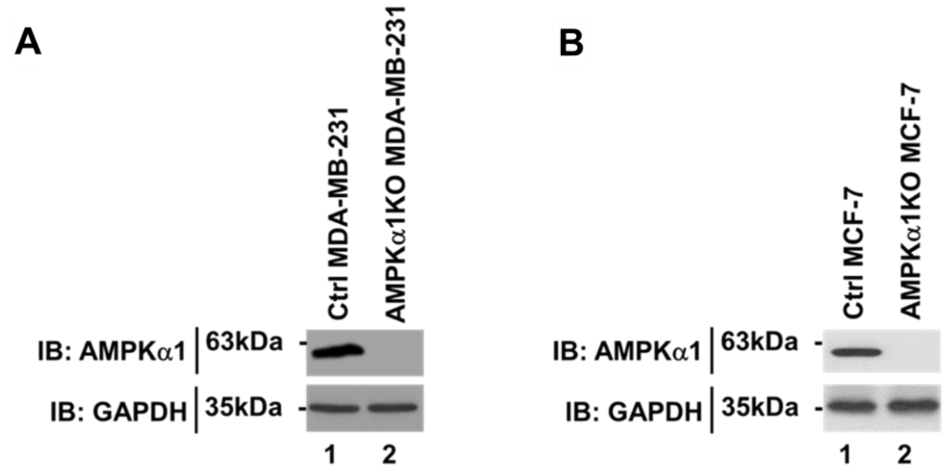

Figure S5. Generation of *AMPKα1*-knockout MDA-MB-231 and *AMPKα1*-knockout MCF-7 cells. (**A and B**) AMPKα1-knockout MDA-MB-231 (**A**) and *AMPKα1*-knockout MCF-7 cells (**B**) were generated, as described in supplementary Materials and Methods. The efficacy of *AMPKα1*-knockout was examined by using western blotting with antibodies to AMPKα1 or GAPDH as loading control. Uncropped western blot images available in Supplementary Figure S14.

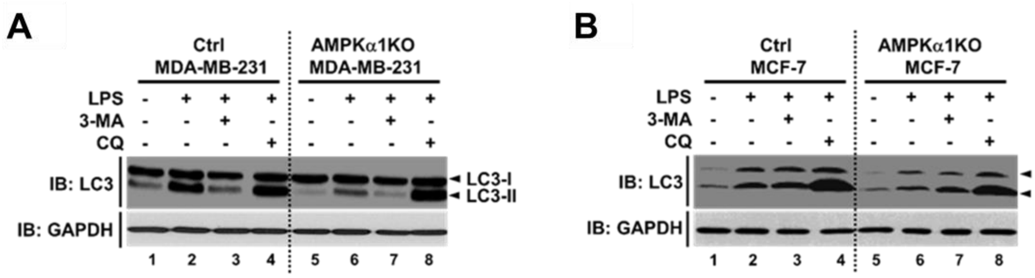

Figure S6. Suppression of autophagy in *AMPKα1*-knockout MDA-MB-231 and *AMPKα1*-knockout MCF-7 cells. (**A and B**) Ctrl MDA-MB-231 and *AMPKα1*-knockout MDA-MB-231 (**A**), or Ctrl MCF-7 and *AMPKα1*-knockout MCF-7 cells (**B**) were treated with or without 3-MA (5mM), or CQ (10 μM), in the presence or absence of LPS (10 μg/ml), for 6 h. Whole cell lysates were immunoblotted with anti-LC3 antibody and anti-GAPDH as a loading control. Uncropped western blot images available in Supplementary Figure S15.

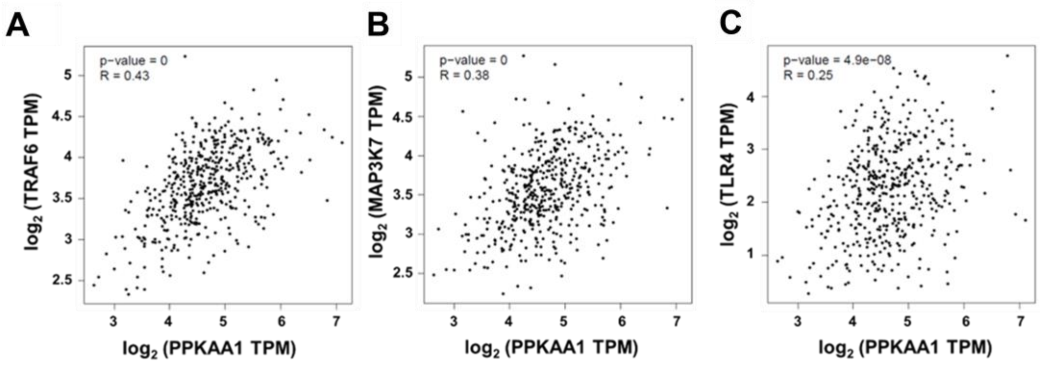

Figure S7. Correlation between AMPKαl (*PPKAA1*) and TRAF6 (**A**), TAK1 (*MAP3K7*) (**B**), or TLR4 (**C**) in LUAD cancers revealed by GEPIA

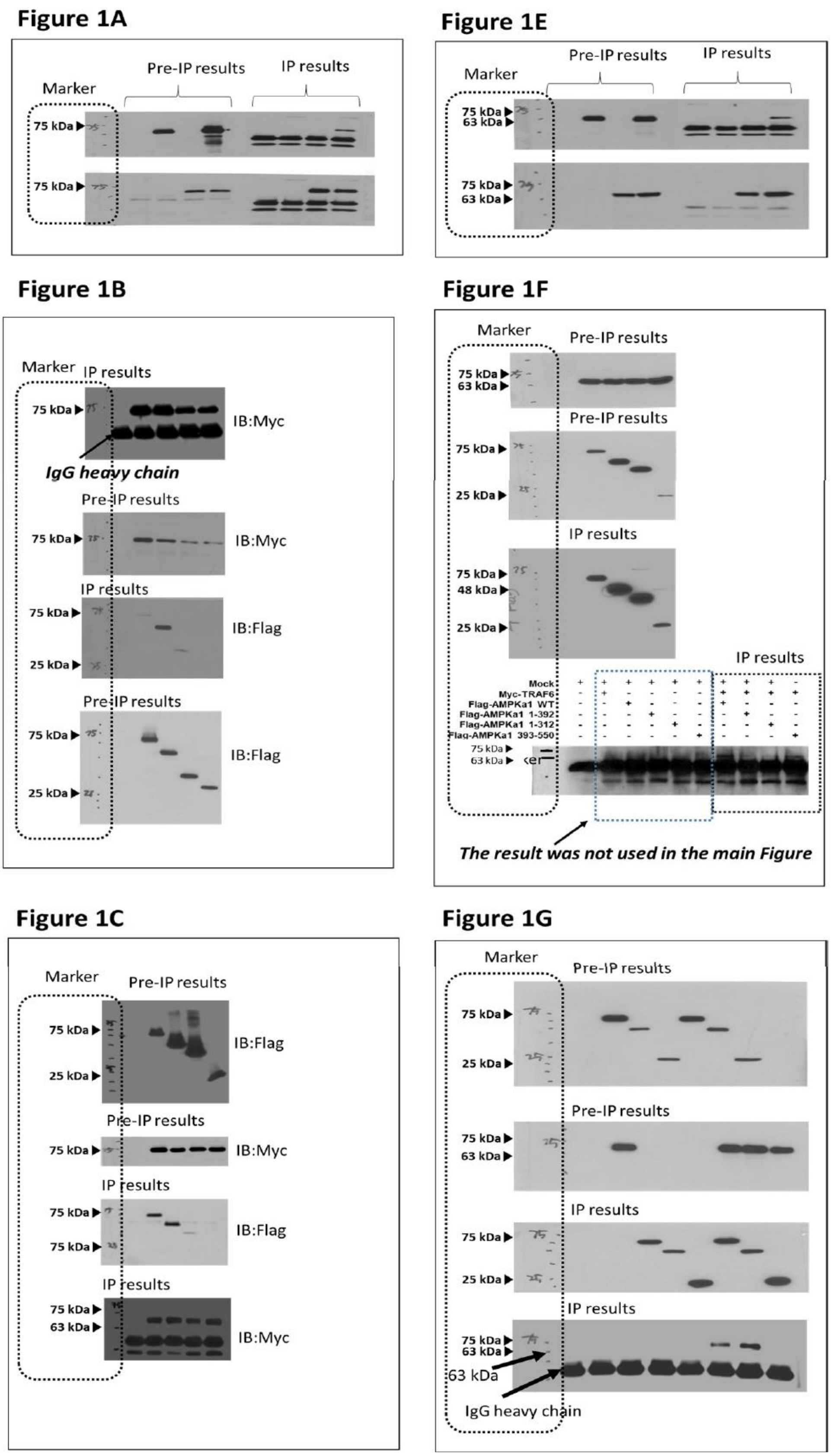

Figure S8. Uncropped western blot images for Figure 1.

Figure 2B

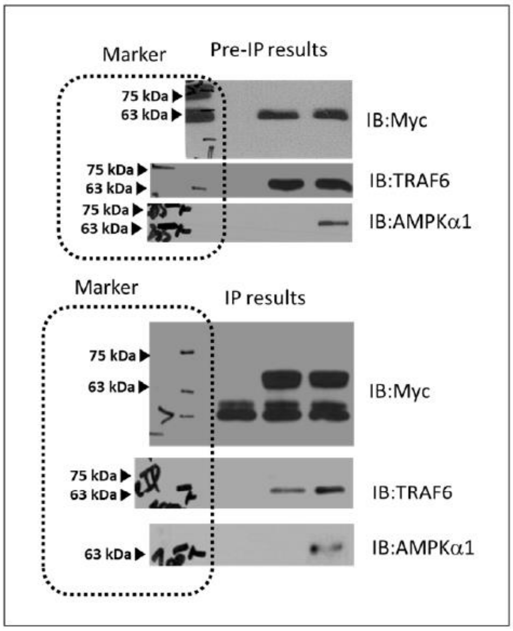

Figure 2C

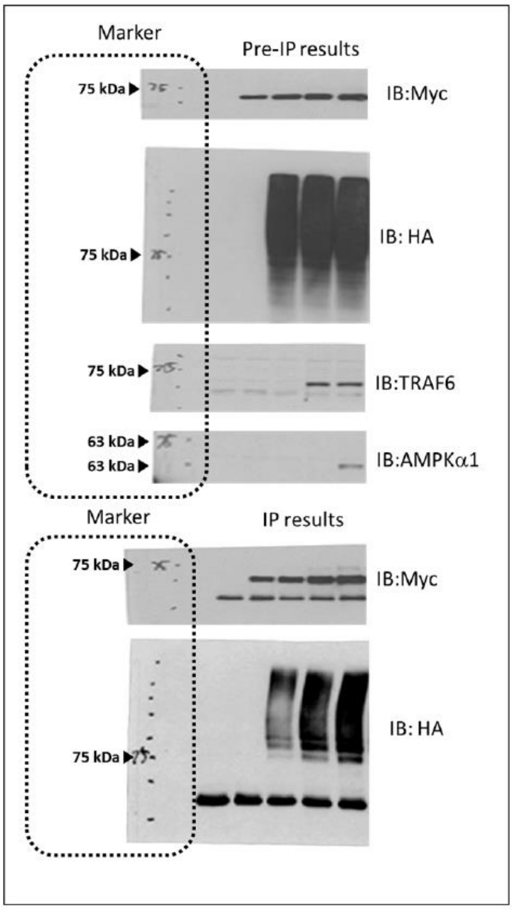

Figure 2D

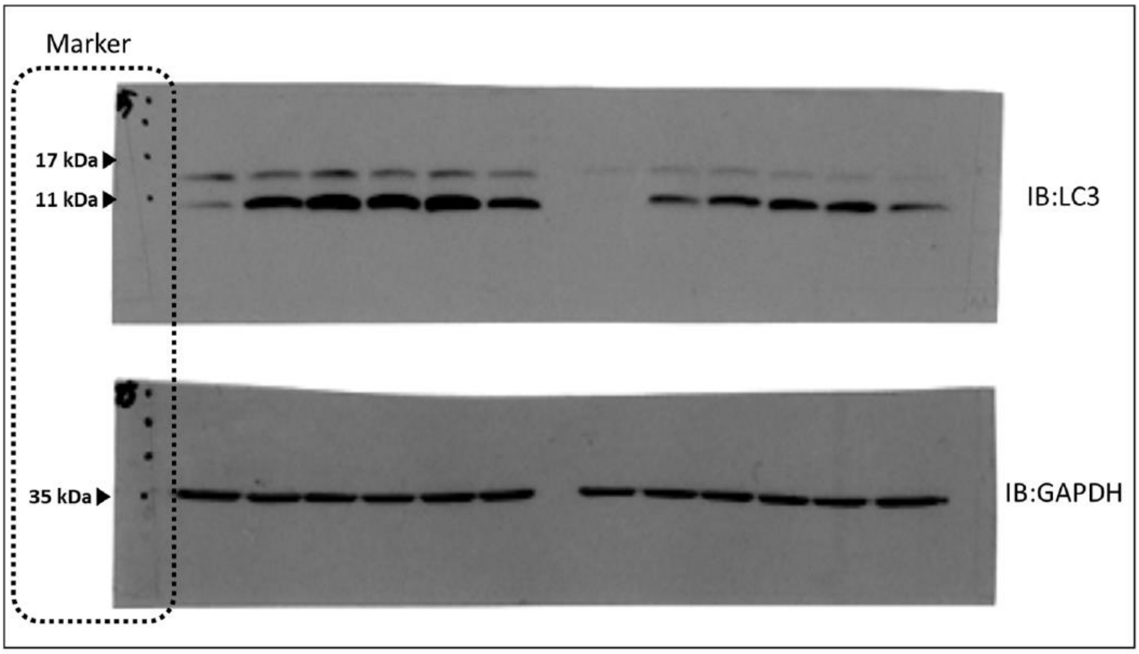

Figure S9. Uncropped western blot images for Figure 2.

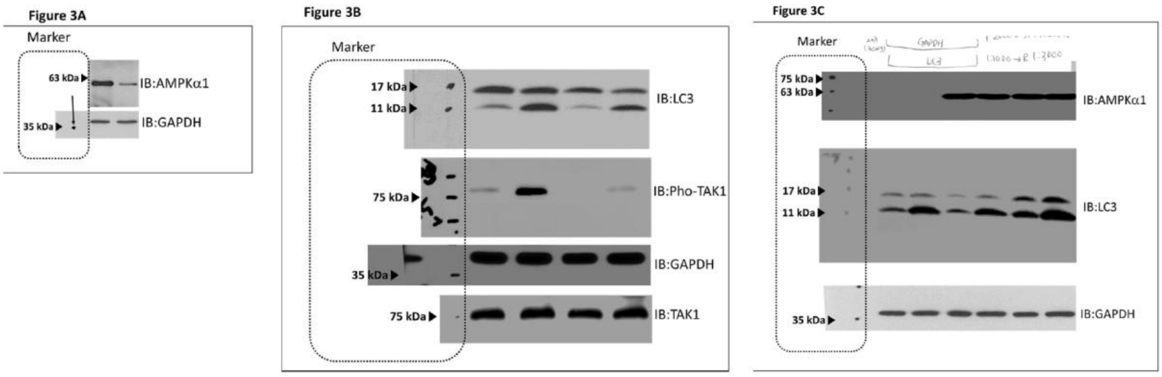

Figure S10. Uncropped western blot images for Figure 3.

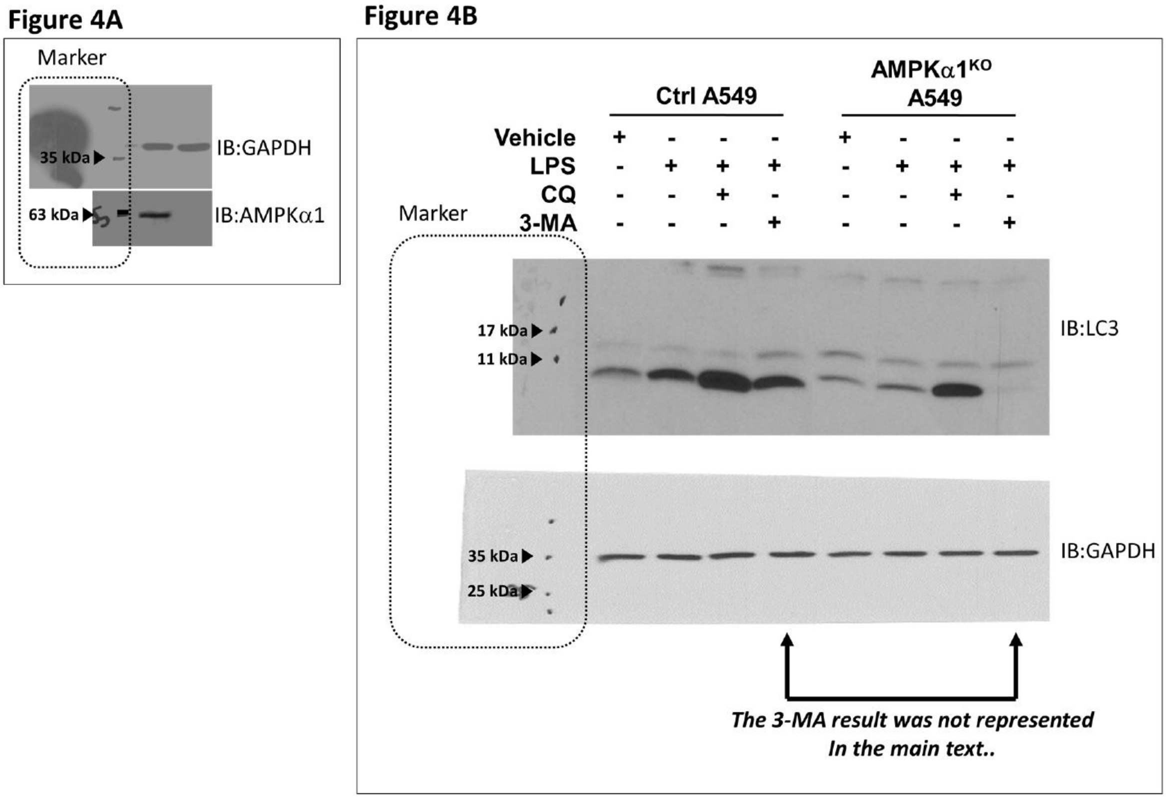

Figure S11. Uncropped western blot images for Figure 4.

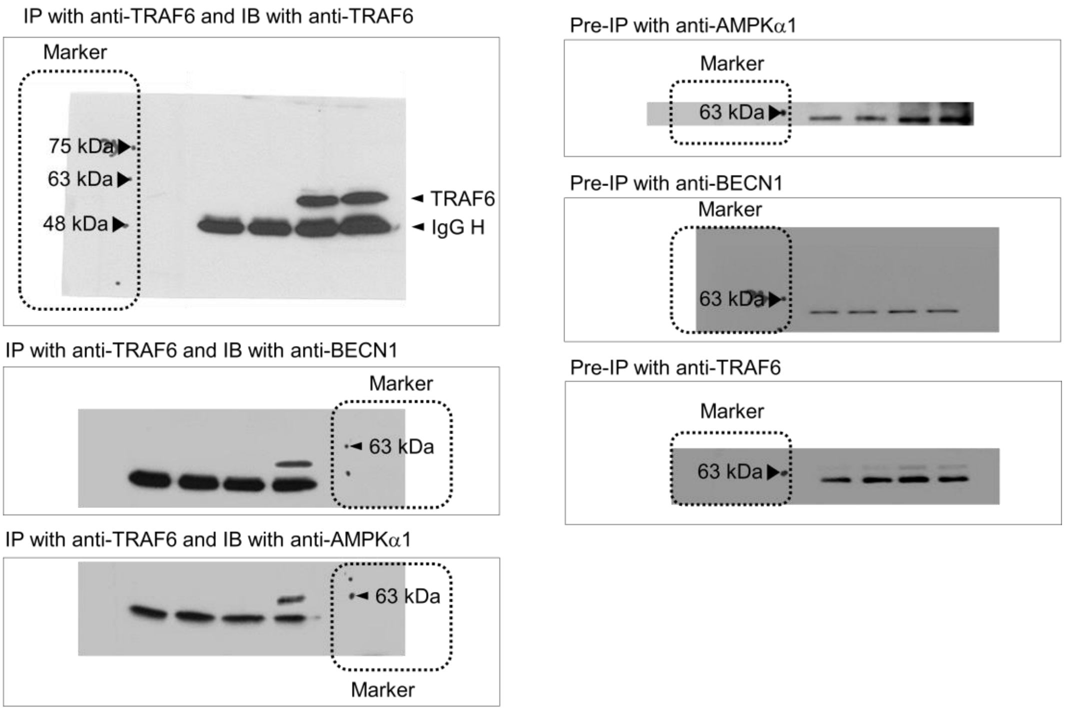

Figure S12. Uncropped western blot images for Supplementary Figure S2.

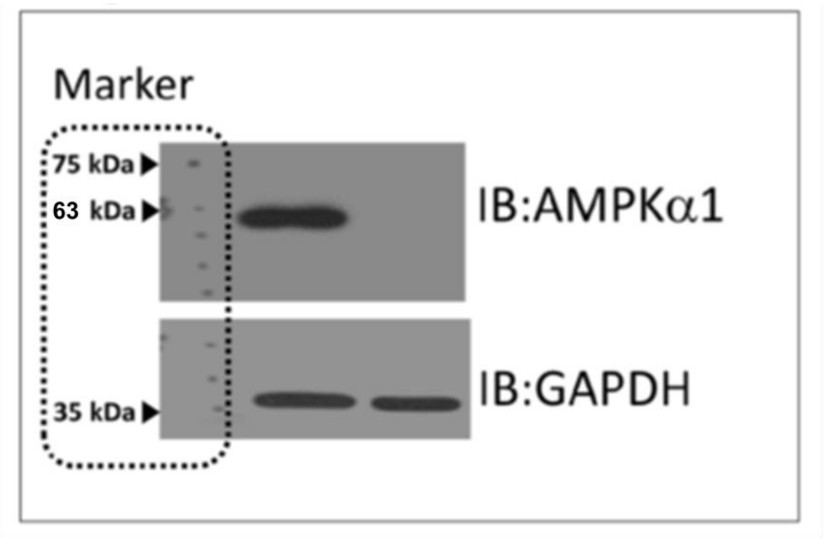

Figure S13. Uncropped western blot images for Supplementary Figure S3.

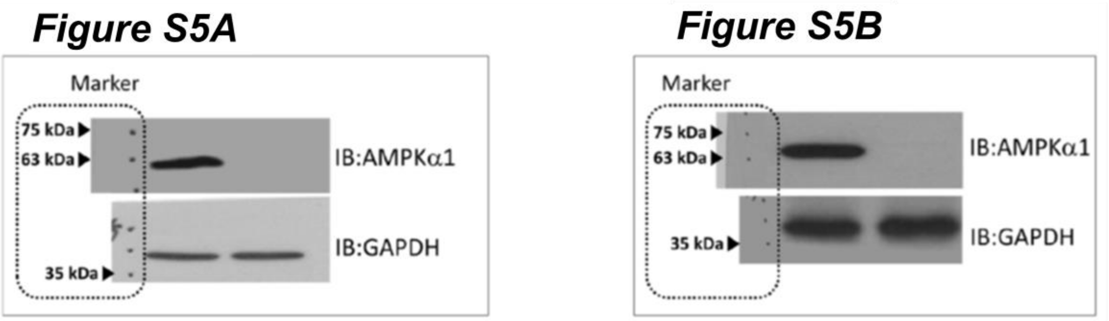

Figure S14. Uncropped western blot images for Supplementary Figure S5.

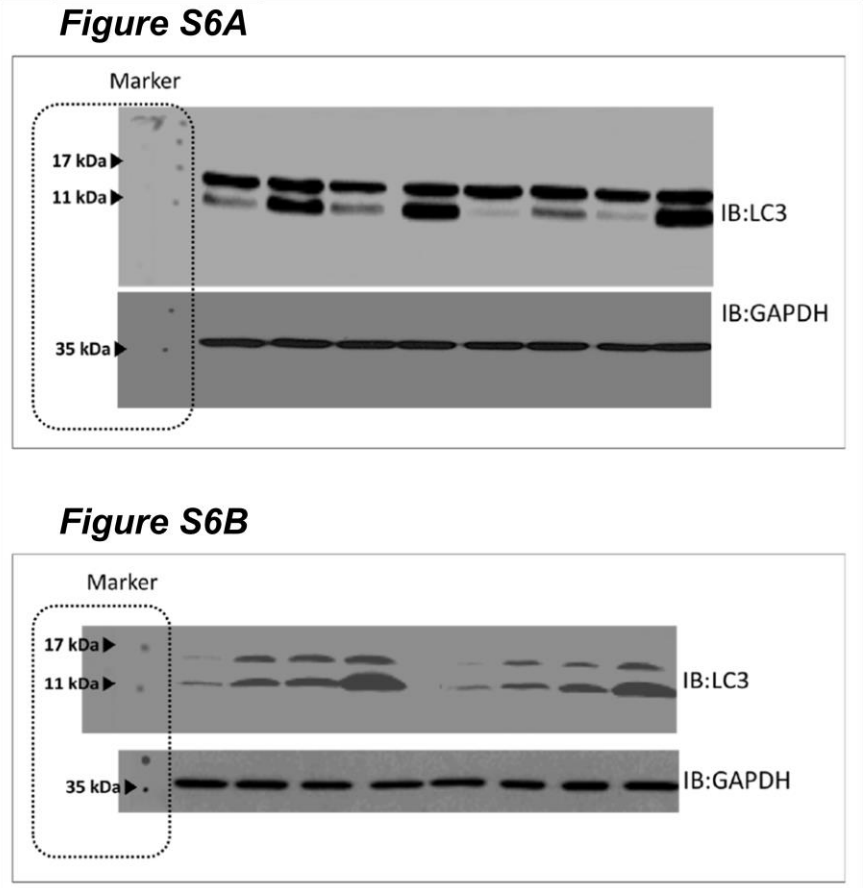

Figure S15. Uncropped western blot images for Supplementary Figure S6.

Reference

1. Kim, S.Y.; Jeong, S.; Jung, E.; Baik, K.-H.; Chang, M.H.; Kim, S.A.; Shim, J.-H.; Chun, E.; Lee, K.-Y. AMP-activated protein kinase-α1 as an activating kinase of TGF-β-activated kinase 1 has a key role in inflammatory signals. *Cell Death Dis.* **2012,** *3*, e357.
2. Min, Y.; Kim, M.J.; Lee, S.; Chun, E.; Lee, K.Y. Inhibition of TRAF6 ubiquitin-ligase activity by PRDX1 leads to inhibition of NFKB activation and autophagy activation. *Autophagy* **2018**, *14*, 1347–1358.
3. Min, Y.; Lee, S.; Kim, M.J.; Chun, E.; Lee, K.Y. Ubiquitin-Specific Protease 14 Negatively Regulates Toll-Like Receptor 4-Mediated Signaling and Autophagy Induction by Inhibiting Ubiquitination of TAK1-Binding Protein 2 and Beclin 1. *Front. Immunol.* **2017**, *8*, 1827.
4. Kim, M.J.; Min, Y.; Im, J.S.; Son, J.; Lee, J.S.; Lee, K.Y. p62 is Negatively Implicated in the TRAF6-BECN1 Signaling Axis for Autophagy Activation and Cancer Progression by Toll-Like Receptor 4 (TLR4). *Cells* **2020**, *9*, 1142.
5. Min, Y.; Wi, S.M.; Shin, D.; Chun, E.; Lee, K.Y. Peroxiredoxin-6 Negatively Regulates Bactericidal Activity and NF-κB Activity by Interrupting TRAF6-ECSIT Complex. *Front. Cell Infect. Microbiol*. **2017**, *7*, 94.
6. Min, Y.; Wi, S.M.; Kang, J.A.; Yang, T. Cereblon negatively regulates TLR4 signaling through the attenuation of ubiquitination of TRAF6. *Cell Death Dis.* **2016,** *7*, e2313.
7. Kim, M.J.; Min, Y.; Shim, J.H.; Chun, E.; Lee, K.Y. CRBN Is a Negative Regulator of Bactericidal Activity and Autophagy Activation Through Inhibiting the Ubiquitination of ECSIT and BECN1. *Front. Immunol.* **2019**, *10*, 2203.

© 2020 by the authors. Licensee MDPI, Basel, Switzerland. This article is an open access article distributed under the terms and conditions of the Creative Commons Attribution (CC BY) license (http://creativecommons.org/licenses/by/4.0/).
